# Role of Alanine Dehydrogenase of *Mycobacterium tuberculosis* during Recovery from Hypoxic Nonreplicating Persistence

**DOI:** 10.1371/journal.pone.0155522

**Published:** 2016-05-20

**Authors:** Michelle M. Giffin, Lanbo Shi, Maria L. Gennaro, Charles D. Sohaskey

**Affiliations:** 1 Department of Veterans Affairs Medical Center, Long Beach, CA, United States of America; 2 Public Health Research Institute, New Jersey Medical School, Rutgers University, Newark, NJ, United States of America; University of Freiburg, GERMANY

## Abstract

*Mycobacterium tuberculosis* can maintain a nonreplicating persistent state in the host for decades, but must maintain the ability to efficiently reactivate and produce active disease to survive and spread in a population. Among the enzymes expressed during this dormancy is alanine dehydrogenase, which converts pyruvate to alanine, and glyoxylate to glycine concurrent with the oxidation of NADH to NAD. It is involved in the metabolic remodeling of *M*. *tuberculosis* through its possible interactions with both the glyoxylate and methylcitrate cycle. Both mRNA levels and enzymatic activities of isocitrate lyase, the first enzyme of the glyoxylate cycle, and alanine dehydrogenase increased during entry into nonreplicating persistence, while the gene and activity for the second enzyme of the glyoxylate cycle, malate synthase were not. This could suggest a shift in carbon flow away from the glyoxylate cycle and instead through alanine dehydrogenase. Expression of *ald* was also induced *in vitro* by other persistence-inducing stresses such as nitric oxide, and was expressed at high levels *in vivo* during the initial lung infection in mice. Enzyme activity was maintained during extended hypoxia even after transcription levels decreased. An *ald* knockout mutant of *M*. *tuberculosis* showed no reduction in anaerobic survival *in vitro*, but resulted in a significant lag in the resumption of growth after reoxygenation. During reactivation the *ald* mutant had an altered NADH/NAD ratio, and alanine dehydrogenase is proposed to maintain the optimal NADH/NAD ratio during anaerobiosis in preparation of eventual regrowth, and during the initial response during reoxygenation.

## Introduction

*Mycobacterium tuberculosis* is able to persist in humans for decades producing latent (asymptomatic) tuberculosis. Sequestered in granulomas, bacteria survive by entering a nonreplicating persistent state [1,2]. Reactivation followed by active disease can occur, with the risk estimated at 5–10% over a lifetime. Tuberculous granulomas can be hypoxic in humans, maintaining oxygen levels that prevent *M*. *tuberculosis* from replicating [2–5]. Since *M*. *tuberculosis* is an obligate aerobe, hypoxia presents many challenges including energy production and redox balancing.

During adaptation to decreasing oxygen levels, *M*. *tuberculosis* undergoes extensive metabolic remodeling which allows it to survive anaerobiosis. For example the bacteria express the less energy efficient cytochrome *bd* oxidase which has high affinity for oxygen, and shunts electron flow through the non-proton pumping type II NADH dehydrogenase [6]. A decreasing flow of electrons due to the lack of oxygen is associated with an increase in the concentration of reduced cofactors, such as NADH [2,7,8]. Nitrate can be utilized as an alternative electron acceptor in place of oxygen [9,10]. Changes that occur during reactivation, when oxygen levels increase to a level sufficient to support replication of *M*. *tuberculosis*, are largely unknown.

One of the genes upregulated in response to hypoxia is *ald* encoding alanine dehydrogenase (Ald), which is essential for the utilization of alanine as a nitrogen source [11]. Unlike Ald of some other bacteria, Ald of *M*. *tuberculosis* is a multispecific enzyme using either glyoxylate or pyruvate as substrates [11]. It catalyzes the reduction of pyruvate to alanine with pyruvate reductive aminase activity (PvRA), or glyoxylate to glycine with glyoxylate reductive aminase activity (GxRA) coupled with the oxidation of NADH to NAD. In the reverse direction it oxidizes alanine to pyruvate (ALD), but does not use glycine as a substrate [11]. *In vivo* alanine dehydrogenase enzymes have been shown to catalyze the production of alanine or glycine [12,13].

**Fig 1:**
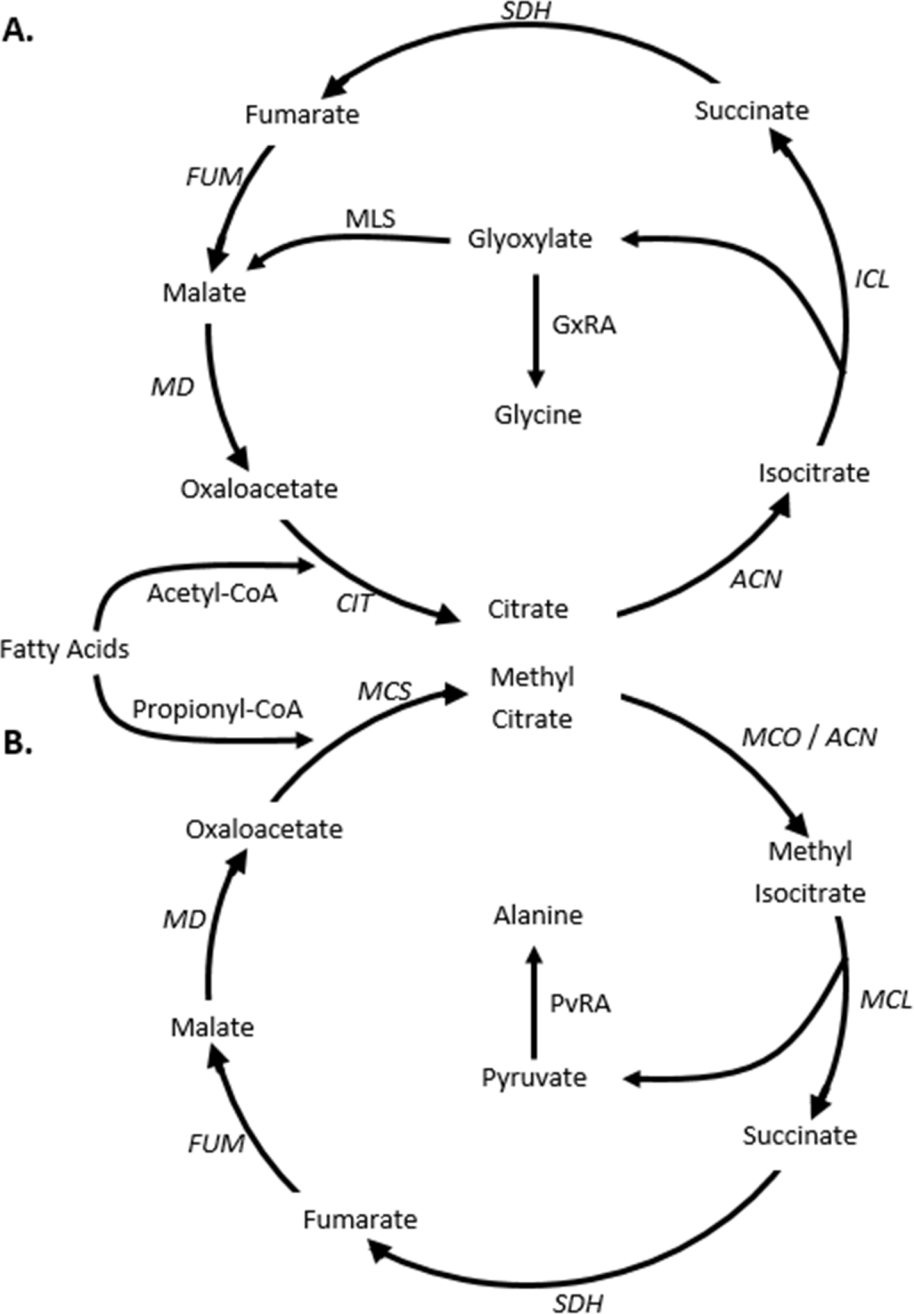
Interaction of Ald with two metabolic pathways. Even-chain fatty acids are degraded to acetyl-CoA while odd-chain fatty acids also produce propionyl-CoA. Acetyl-CoA is oxidized by the (A) glyoxylate cycle and propionyl-CoA by the (B) methylcitrate cycle. Enzyme activities are indicated in italics. ACN–aconitase; ICL–isocitrate lyase; MLS–malate synthase; MD–malate dehydrogenase; CIT–citrate synthase; SDH–succinate dehydrogenase; FUM–fumarase; MCD–methylcitrate dehydratase; MCL methylisocitrate lyase; MCS–methylcitrate synthase; GxRA–glycine dehydrogenase; PvRA–alanine dehydrogenase. ICL and MCL activities are catalyzed by the same isocitrate lyase enzyme. GxRA and PvRA activities are produced by the same alanine dehydrogenase.

It has been proposed that alanine dehydrogenase plays a role in maintaining redox balance during shiftdown of *M*. *tuberculosis* to the NRP state [1,14]. Decreasing oxygen results in a shift in the NADH/NAD ratio towards NADH. The two substrates for alanine dehydrogenase, pyruvate and glyoxylate, would be supplied by isocitrate lyase, the first unique enzyme of the glyoxylate and the methylcitrate cycles (Fig 1). The anaplerotic glyoxylate pathway is used to synthesize C_4_ compounds from the acetyl-CoA produced during β-oxidation of fatty acids, which are the main sources of energy for *M*. *tuberculosis in vivo* (reviewed in [15]). The methylcitrate cycle is utilized during metabolism of odd-chain fatty acids and cholesterol, where propionyl-CoA is produced [16] (Fig 1). In *M*. *tuberculosis* isocitrate lyase is a multispecific enzyme as it serves also as the 2-methylisocitrate lyase which converts methylisocitrate to pyruvate and succinate [17]. The interaction of alanine dehydrogenase with either of these two cycles is uncharacterized.

Here alanine dehydrogenase and its interaction with the glyoxylate cycle were investigated. After the initial induction of both isocitrate lyase and alanine dehydrogenase, the former was down regulated while the latter was further upregulated. The late expression of Ald, and a defect in regrowth of an Ald mutant of *M*. *tuberculosis* suggested a role for Ald during reaeration. It is proposed that Ald is involved in maintaining the optimal NADH/NAD ratio not only during dormancy, but also during reactivation when oxygen levels increase enough to support regrowth of *M*. *tuberculosis*.

## Results

### Expression of genes and enzymes of the glyoxylate cycle

Alanine dehydrogenase, encoded by *ald*, has glyoxylate reductive aminase activity (GxRA), which could constitute a branch from the glyoxylate cycle [1,14] (Fig 1). The enzymes unique to the glyoxylate cycle are the two isocitrate lyases (Icl), encoded by *icl1* and *icl2*, and malate synthase encoded by *glcB*. To determine how alanine dehydrogenase of *M*. *tuberculosis* H37Rv interacts with this cycle the expression of these genes was determined in *in vitro* and *in vivo* models.

We used the Wayne model as the *in vitro* model for dormancy [18]. Growth of *M*. *tuberculosis* cultures in sealed tubes results in gradual depletion of oxygen from the medium, giving rise to first a microaerobic nonreplicating persistent phase 1 (NRP-1) followed by the anaerobic NRP-2. In the latter stage *M*. *tuberculosis* can survive for extended periods of time.

**Fig 2:**
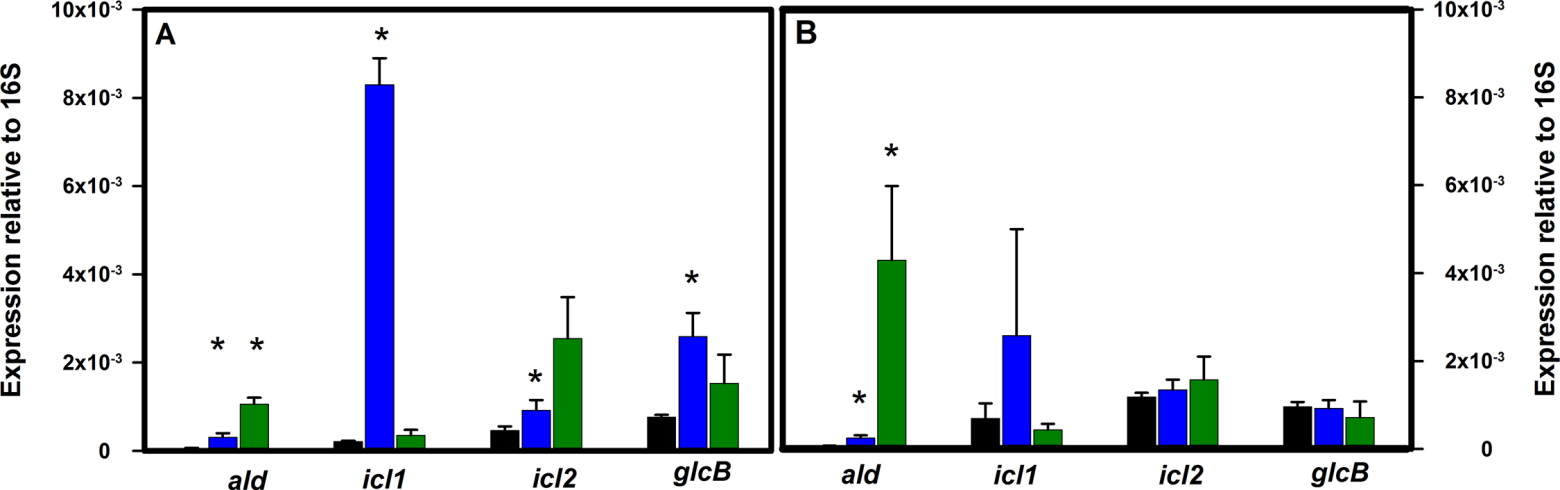
Expression of genes involved in the glyoxylate cycle. Total RNA from *M*. *tuberculosis* cultures in either aerobic; microaerobic NRP-1 or anaerobic NRP-2 was extracted and quantitation of transcripts performed. (A) H37Rv. (B) Erdman. Black bars–aerobic cultures. Blue bars–NRP-1, and green bars–NRP-2. RNA levels are expressed relative to the stable 16S rRNA. The standard deviation is shown. Asterisks indicate statistical significance (p<0.05) of the comparison with the aerobic samples.

Expression of *ald* in the Wayne model was induced 5-fold in microaerobic NRP-1, but the highest expression was in anaerobic NRP-2 (16-fold induction relative to mid-log aerobic growing cultures) (Fig 2A). *icl1* was strongly induced in NRP-1 (39-fold) but expression then decreased in NRP-2. *icl2* showed a similar pattern to *ald* with the highest induction in NRP-2. *glcB* was induced 3-fold in NRP-1.

**Fig 3:**
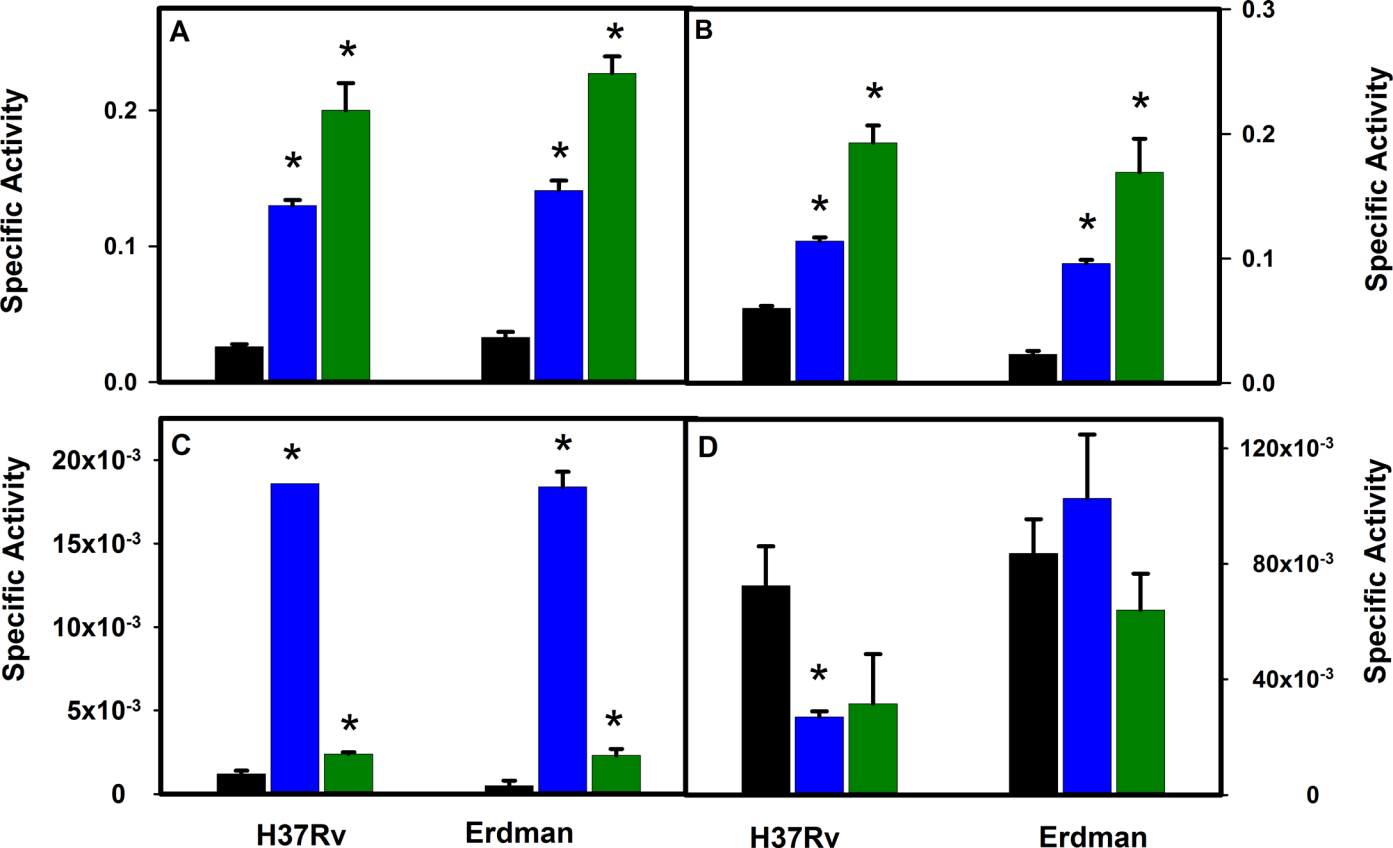
Enzyme Activities in *M*. *tuberculosis* H37Rv and Erdman. A: Glyoxylate reductive aminase activity. B: pyruvate reductive aminase activity. C:isocitrate lyase activity. D: malate synthase activity. Black bars are aerobic cultures, blue bars are NRP-1 and green bars are NRP-2. All units are μmoles/min/mg protein. The standard deviation is shown. Asterisks indicate statistical significance (p<0.05) of the comparison with the aerobic samples.

To verify that transcription levels reflected protein levels, enzyme activities were measured in aerobic, NRP-1 and NRP-2 cultures (Fig 3). Both GxRA and PvRA activities were induced by hypoxia. GxRA was induced 5-fold and PvRA 2-fold in NRP-1, while GxRA increased 8-fold, and PvRA 3-fold in NRP-2. Both showed peak activity in NRP-2 and, although activity levels were not identical, they closely reflected transcription changes. Isocitrate lyase was most active in NRP-1 (16-fold induction), which also closely mirrored transcription changes. Malate synthase levels decreased, which could suggest an additional mechanism of regulation.

The *icl2* gene of *M*. *tuberculosis* H37Rv produces an inactive enzyme due to a frameshift mutation. To exclude the possibility that this altered the expression of other genes, transcription was measured in *M*. *tuberculosis* Erdman, which produces an active Icl2 enzyme (Fig 2B). Results with the Erdman strain were similar to those obtained with H37Rv. *ald* was induced 4-fold in NRP-1 and 64-fold in NRP-2 relative to aerobic growing cultures. GxRA and PvRA levels increased in NRP-1 (both 4-fold relative to aerobic growing cultures) and 7-fold in NRP-2 cultures (Fig 3). Activities of enzymes reflected the increase in transcription although GxRA and PvRA levels did not fully reflect the induction in transcription in NRP-2. *icl1* was induced in NRP-1 (4-fold) however not to the extent observed in H37Rv, but then decreased in NRP-2 similar to H37Rv. The increase in isocitrate lyase activity in NRP-1 in the Erdman strain (35-fold) was similar to that seen in H37Rv. Activity then decreased, although not to the extent that was detected in H37Rv, indicating that Icl2 contributes little activity under these conditions. Malate synthase transcription was not induced in either NRP-1 or NRP-2, and enzyme activities remained relatively constant.

In conclusion, both RNA and enzyme levels of alanine dehydrogenase and isocitrate \\lyase were induced by hypoxia, while malate synthesis was unaffected or decreased. Isocitrate lyase showed the strongest induction during microaerobic NRP-1 and then declined, while alanine dehydrogenase peaked in anaerobic NRP-2.

### Ald expression *in vivo*, and the response to nitric oxide exposure and hypoxia *in vitro*

**Fig 4:**
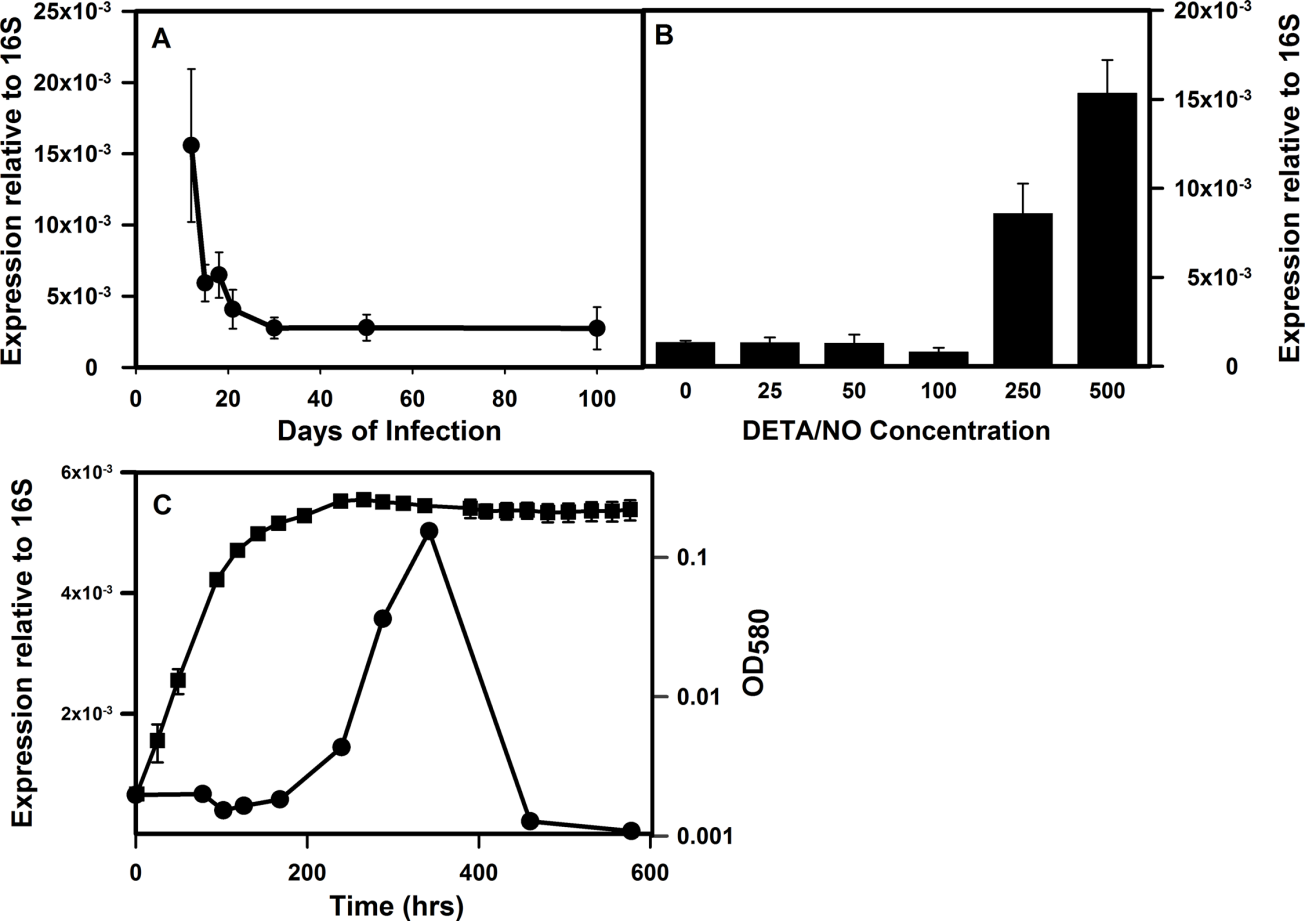
Transcriptional analysis of *ald in vivo*, and in response to stress conditions *in vitro*. (A) Expression of *ald* in the lungs of mice. Lungs were harvested from mice at the indicated time points post-infection. Total RNA was extracted and quantitation of bacterial transcripts was performed. Shown is the mean (± standard deviation) of normalized mRNA copy numbers relative to 16S rRNA from three mice at each time point. (B) Expression after exposure to the NO donor DETA/NO. (C) Expression of *ald* during extended incubation in the Wayne model. Circles indicate expression of *ald* relative to the 16S RNA, and squares indicate the optical density of the culture at 580 nm.

We had previously measured the expression of *icl1* and *glcB* in the lungs of mice over the course of chronic infection [19]. Transcription of *icl1* showed a peak in the initial stages of infection, and then decreased during the chronic stage, while *glcB* transcription declined following the initial infection. The expression of *ald* by *M*. *tuberculosis* in the lungs of mice was determined. Expression of *ald* was high during the initial stage of the infection, and then downregulated with progression of the infection (Fig 4A). The different expression pattern of *ald* during *in vivo* infection in comparison to that of *in vitro* models may reflect the lack of hypoxia in infected mouse lungs [2,3].

In mice the production of nitric oxide is essential for the control of *M*. *tuberculosis*. Although its role in humans is less clear, it is produced by macrophages following infection with *M*. *tuberculosis* [20]. Many genes induced in response to hypoxia are also regulated by nitric oxide. Therefore the expression of *ald* during *in vitro* exposure to NO was tested. *ald* was strongly induced by the NO donor DETA/NO at levels above 100 mg/ml (Fig 4B).

The expression of *ald* during oxygen limitation was examined in more detail (Fig 4C). In the Wayne model cultures become microaerobic (NRP-1) by 100 hrs, and are anaerobic by 250 hrs (NRP-2). It was found that *ald* was fully induced by NRP-2 reaching a peak around 350 hrs in the Wayne model before declining.

### Role of Ald in recovery from extended hypoxia

The survival of the *ald* deletion mutant RVW7 was analyzed during extended hypoxia in the Wayne model (S1 Fig). In comparison to wild type (WT) no difference in survival was detected. However it was noticed that RVW7 colonies were slower to appear on DOA plates, although they eventually reached numbers similar to wild type. This, along with the late expression of *ald* in the Wayne model, suggested a possible role for Ald in recovery, rather than entry into, hypoxic NRP.

However by 600 hr, *ald* transcription is low (Fig 4C). To determine whether enzyme activities were reflective of transcription changes, GxRA, and PvRA activities were determined at 350 and 600 hrs. Despite the decline in transcription of *ald* after 350 hrs, GxRA activity remained essential constant (0.15 U ± 0.06, at 350 hrs and 0.19 ± 0.09 after 600 hrs). PvRA activities also did not change significantly over this time frame (0.23 U ± 0.09 at 350 hrs, and 0.19 ± 0.03 at 600 hrs) suggesting Ald is stably maintained even after transcription decreases.

**Fig 5:**
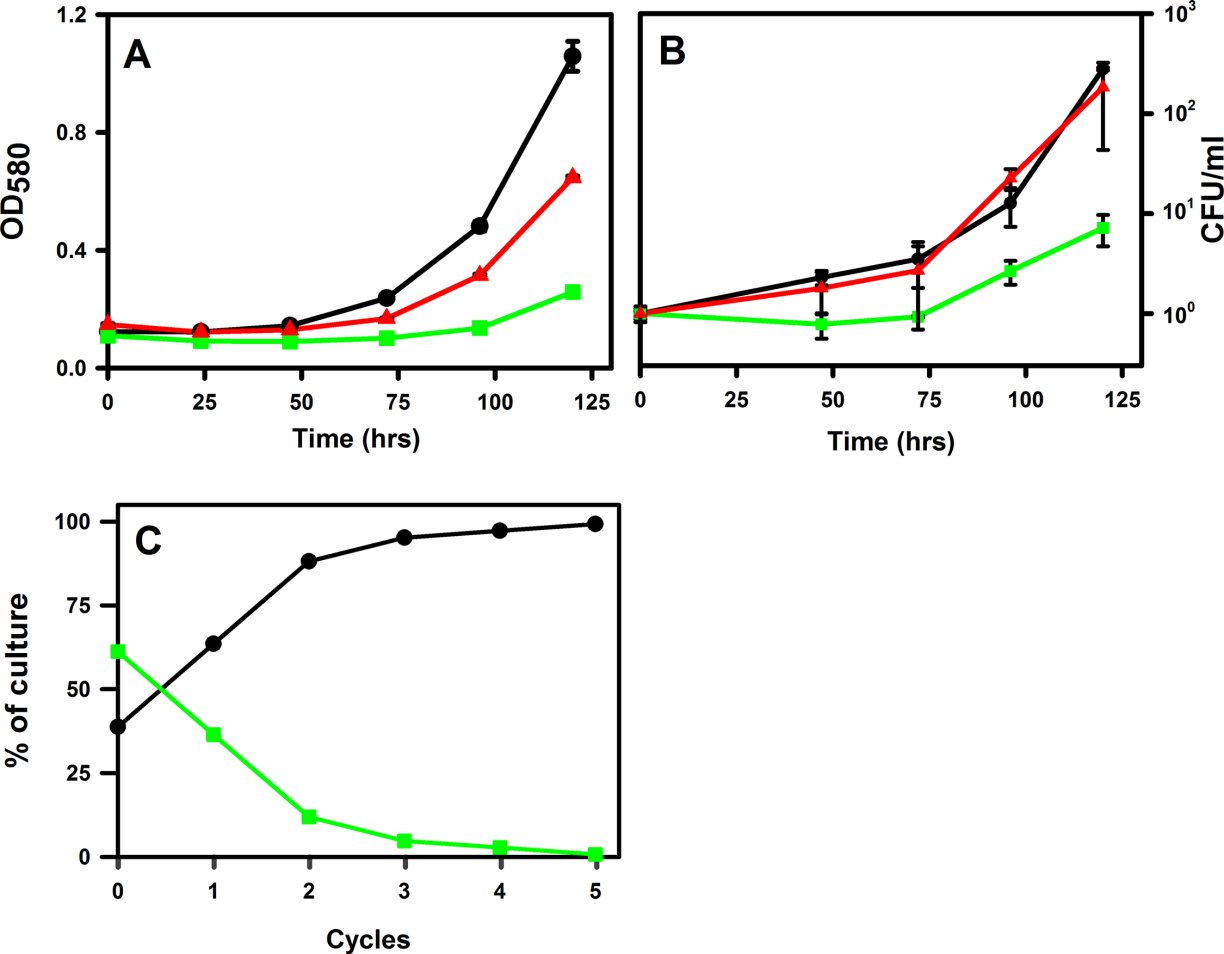
Regrowth dynamic of *M*. *tuberculosis* during recovery from hypoxia. Cultures were grown for 600 hrs in the Wayne model, diluted in fresh media and then grown with vigorous shaking. Growth was monitored by (A) optical density at 580 nm or (B) plating to determine CFUs. (C) Growth competition assay. WT and RVW7 were mixed in equal concentration and subjected to repeated cycles in the Wayne model. CFUs were determined after each cycle. WT–black circles. RVW7 –green squares. RVW7 pAld-Gen21 –red triangles.

To test for a possible role during reaeration the growth of the *ald* mutant was analyzed. Cultures of wild type *M*. *tuberculosis*, RVW7 and the complemented mutant RVW7 pAld-Gen21 were grown in the Wayne model. There were no differences in growth rate and shiftdown curves between strains (data not shown). After 600 hrs (approximately 400 hrs of anaerobic conditions) cultures were opened and diluted 1:2 in fresh medium. Relative to wild type, the RVW7 mutant showed a reproducible and significant delay in growth as determined by optical density (Fig 5A). This was not seen in the complemented mutant. The delay was also observed when CFU’s were measured (Fig 5B).

The survival of RVW7 in competition with wild type was determined during several cycles of hypoxia. Both strains were grown together for 600 hrs in the Wayne model, plated to measure CFUs, and then diluted into fresh DTA for continued incubation. After growth in broth the culture was again inoculated into the Wayne model. In each passage the percentage of the *ald* mutant in the culture decreased while the WT strain increased (Fig 5C). After four passages the wild type strain constituted greater than 95% of the culture indicating a significant defect in reactivation in the *ald* mutant.

### Role of Ald in redox balancing

A possible function for Ald during hypoxia is redox homeostasis [1]. With decreasing levels of oxygen *M*. *tuberculosis* experiences a shift in the NADH/NAD ratio towards elevated NADH [4,7,8,21]. Ald could perform a role in adjusting this ratio by the conversion of glyoxylate to glycine, and/or pyruvate to alanine. During fermentative growth of *E*. *coli*, this function is performed mainly by lactate dehydrogenase by converting pyruvate to lactate while oxidizing NADH to NAD. To determine whether Ald could maintain redox balance during anaerobiosis, the ability of the *M*. *tuberculosis ald* to complement a lactate dehydrogenase mutant (*ldh*) of *E*. *coli* was tested. The *ald* gene from *M*. *tuberculosis* was cloned under the control of the *ldh* promoter, integrated into the *ldh* locus of *E*. *coli* SZ194. As a negative control a hygromycin marker was integrated into *ldh*. SZ194 *ldh*::*hyg* did not grow anaerobically while SZ194 *ldh*::*ald* did (S2 Fig) suggesting Ald is able to perform NADH/NAD recycling during anaerobiosis.

### Physiological factors during reaeration

**Fig 6:**
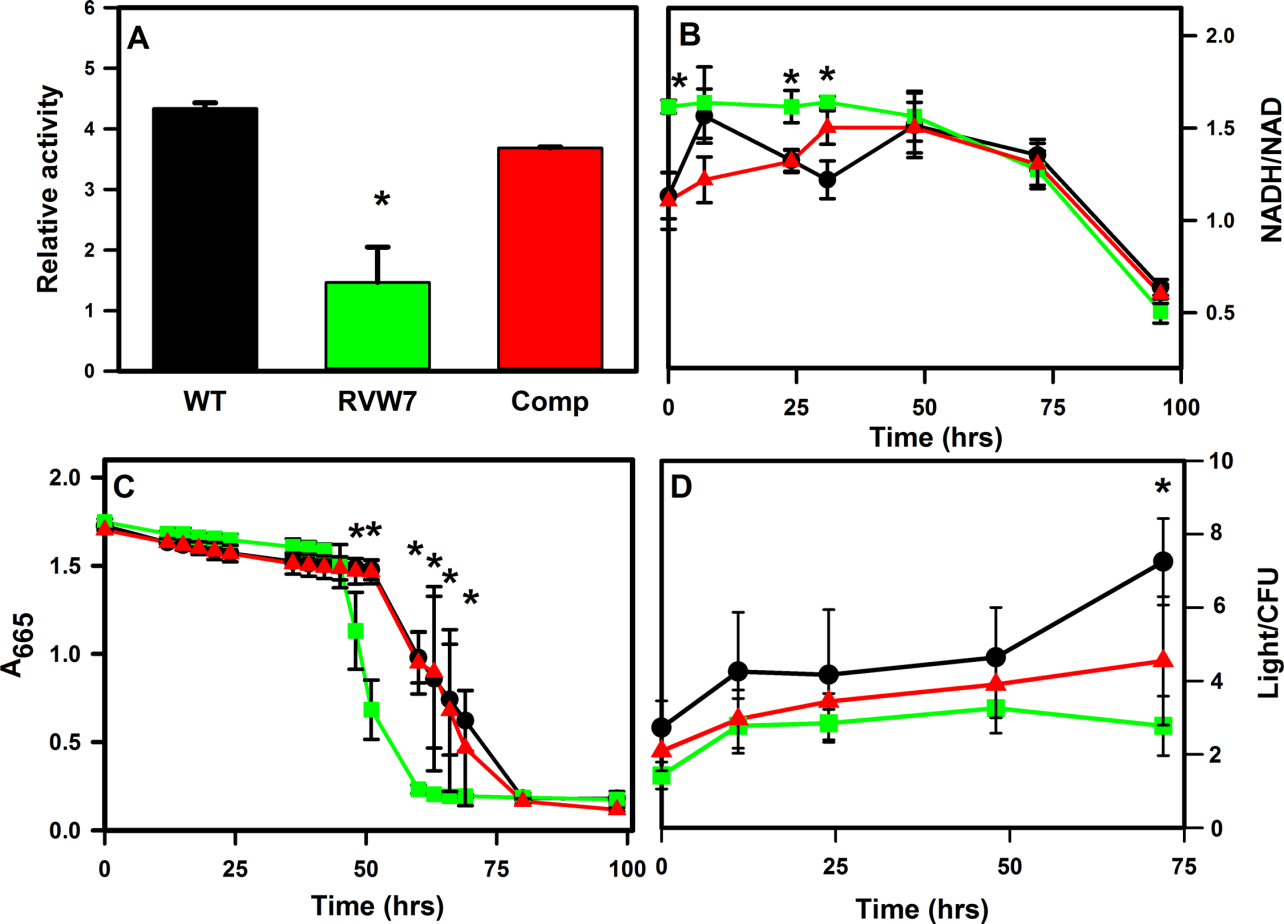
Physiological parameters of *M*. *tuberculosis* during reaeration. (A) Metabolic activity after extended hypoxia. Reducing activity was determined with XTT. Data are shown in arbitrary units. (B) The ratio of NADH to NAD. (C) Oxygen consumption was measured by methylene blue decolorization. (D) ATP levels in arbitrary units normalized to cell number. Black circles–WT, green squares–RVW7, red triangles–RVW7 pAld-Gen21. Asterisks indicate statistical significance (p<0.05) of the comparison of RVW7 with WT.

We propose a role for Ald in maintaining the NADH/NAD ratio of *M*. *tuberculosis* during recovery from hypoxia when oxygen levels increased enough to trigger exit from the NRP state. We analyzed physiological factors during reaeration by determining the metabolic activity of RVW7 after extended hypoxia. First we tested the reducing power of NRP-2 cultures of *M*. *tuberculosis* using the electron acceptor XTT. A significant decrease in reducing power was observed in the *ald* mutant compared to wild type and the complemented mutant (Fig 6A). This difference was not seen with aerobic cultures with these strains (data not shown).

Next, the NADH/NAD ratio was determined in late NRP-2, and during reaeration in wild type and RVW7. At the end of the extended incubation in the Wayne model the NADH/NAD ratio was significantly lower for wild type than the *ald* mutant (Fig 6B). The ratio in WT remained lower in comparison to RVW7 during the lag phase. The ratio in wild type decreased when growth resumed, while in contrast the ratio in RVW7 was higher, but decreased towards that of wild type before growth began.

The NADH/NAD ratio is sensitive to oxygen levels so methylene blue was used to measure oxygen utilization during reaeration. Methylene blue was added to cultures of *M*. *tuberculosis* in NRP-2 to monitor oxygen levels measured during reactivation. Wild type cultures showed a lag phase characterized by an initial slow fading followed by rapid decolorization (Fig 6C). The *ald* mutant reproducibly decolorized the methylene blue more rapidly than WT, beginning after 42 hrs of incubation, and finishing by 60 hrs in comparison to 51 hrs and 80 hrs respectively for WT. No difference was seen with aerobically grown cultures (data not shown). This increase in oxygen utilization preceded bacterial replication in all strains.

A change in the NADH/NAD ratio could also have an effect on ATP levels. ATP levels were determined during re-growth and were similar in WT and the RVW7 mutant after extended hypoxia (Fig 6D). There was no significant difference in ATP levels in the first 50 hrs before resumption of growth, which occurred earlier for WT than RVW7.

## Discussion

In response to decreasing oxygen, *M*. *tuberculosis* induces a set of dormancy genes that enable this obligate aerobe to adapt to, and survive low oxygen conditions. Included in this set, but often unrecognized, are genes functioning not for anaerobic survival, but to initiate recovery leading to eventual growth. These gene products perform important functions as *M*. *tuberculosis* is transitioning out of dormancy, and preparing to induce new protein synthesis.

The expression of Ald in *M*. *tuberculosis* is similar to that seen with some of the germination proteins of *Bacillus subtilis*. Transcription of the *gerA* operon, for example, is upregulated 2.5 hrs after induction of sporulation, and remains high but begins to decrease at the end of sporulation [22,23]. The protein is maintained in the mature spore, and is required to initiate germination in the presence of alanine. We show that *ald* transcription is upregulated in *M*. *tuberculosis* late in NRP-2 and then decreases as the cells enter the general shutdown characteristic of dormancy. Although transcription of *ald* decreases the protein is stably maintained so that the cell will be ready to reactivate when oxygen levels increase.

Alanine dehydrogenase is a multispecific enzyme [11] and part of the core transcriptome, a set of conserved genes important for virulence. A series of new inhibitors of Ald were shown to be lethal to dormant cultures of *M*. *tuberculosis* verifying the great potential of this target [24]. And Ald has been implicated in resistance of *M*. *tuberculosis* to the second-line antibiotic D-cycloserine used to treat drug resistant tuberculosis [25]. We now show that Ald also plays an unexpected role during reaeration since inactivation of *ald* resulted in a delayed recovery from dormancy (Fig 5).

Alanine dehydrogenase could play numerous roles during the recovery of *M*. *tuberculosis*. The production and/or accumulation of both alanine and glycine are a widely seen response to hypoxia with extensive examples from varied life forms including plants [26–28], protozoa [29,30] and mammals [31] suggesting this is an ancient mechanism used for dealing with hypoxia and reaeration. The production of alanine and glycine may be due to aminotransferases rather than Ald.

1. A role for Ald in carbon storage seems unlikely for *M*. *tuberculosis*, although the role of Ald in nitrogen utilization is known [11,32]. In plants, hypoxia due to waterlogging, leads to the accumulation of intracellular alanine, glycine, and succinate for the storage of carbon and nitrogen that are rapidly utilized after reoxygenation [26–28].
2. The oxidation of pyruvate in the TCA cycle requires oxygen. Ald could reduce the accumulation of intracellular pyruvate thus reducing the demand for the limiting levels of oxygen [26,33]. Lower levels of pyruvate would also reduce the buildup of glycolytic intermediates that are toxic to *M*. *tuberculosis* during hypoxia [34].
3. Both glycine and alanine protect against hypoxic injury in many types of cells and tissues [35,36]. Glycine provides protection during hypoxia and reaeration in rat livers presumably by inhibiting oxidative stress and lipid peroxidation [37].
4. We propose that the main role of Ald during hypoxia and recover is to maintain the redox balance of *M*. *tuberculosis*. NAD is the principal oxidant in the cell and essential for many reactions, so NADH must be rapidly oxidized to prevent inhibition of important processes. The ratio between NAD and NADH is tightly regulated by redundant systems, and in *M*. *tuberculosis* is generally between 1:3 and 1:10 [38–41]. When oxygen levels decrease or aerobic respiration is blocked by NO the ratio of NAD to NADH shifts [4,7,21]. This shift is also seen in *M*. *tuberculosis* in the lungs of mice [39] indicating that redox stress is present *in vivo*.

The addition of alanine, glycine, glyoxylate or pyruvate to the medium did not decrease the recovery time of the *M*. *tuberculosis* mutant after hypoxia suggesting the main function of Ald during reaeration is not the production or detoxification of these compounds (data not shown).

We propose that the ratio of NAD to NADH plays a role in regulating recovery of *M*. *tuberculosis* from hypoxia, and may prevent growth until the ratio achieves a certain level. It may also serve as the signal indicating to the cell the optimal time to transition from a nonreplicating persistent state to active growth. Indeed the *ald* mutant was unable to maintain the optimal ratio during anaerobic stress leaving it unprepared for recovery until the NADH/NAD was corrected. The NADH/NAD ratio remained relatively constant for approximately 48 hrs in WT after the oxygen levels increased to a level sufficient to support the growth of *M*. *tuberculosis* (Fig 6B). As oxygen was then utilized (Fig 6C), the ratio further shifted to a range that allowed growth to resume. However, at the end of NRP-2, the ratio was significantly higher in RVW7 suggesting a role for Ald in shifting the ratio towards the more oxidized form. The *ald* mutant is unable to decrease the ratio in the first 50 hours of reaeration and must utilize oxygen for this function which results in a delay in growth resumption. This proposal is supported by the observation that a DosR mutant which also had an altered NADH/NAD ratio, also showed a delay in recovering from extended hypoxia [21]. This would not solve all the redox problems raised by lack of oxygen, and there are undoubtedly other mechanisms at work.

In the absence of a final electron acceptor, fermentation can be used by *M*. *tuberculosis* to produce ATP [2,4,8]. Alanine dehydrogenase converts pyruvate to alanine while recycling NADH to NAD. In the *ald* mutant there was a difference in this ratio, with higher NADH levels in late NRP-2 relative to wild type (Fig 6). Ald was able to fulfill a redox balancing role as shown in a *ldhA E*. *coli* mutant by restoring anaerobic growth (S2 Fig). *M*. *tuberculosis* does not grow anaerobically but an alanine dehydrogenase mutant of the related bacteria *M*. *smegmatis* lost the ability to grow anaerobically suggesting a role for Ald in redox balancing [42].

Both intracellular and extracellular concentrations of alanine and glycine increased during exposure of *M*. *tuberculosis* to hypoxia [8]. There is evidence of increased levels of alanine in the granulomas of guinea pigs infected with *M*. *tuberculosis* [43]. Levels increased as the infection progressed and hypoxia increased. The mechanism of these changes is still unknown as is the possible involvement of Ald.

Alanine dehydrogenase may also play a role in redox balancing during entry into the nonreplicating persistent state by interacting with the glyoxylate and/or methylcitrate cycle. A main source of carbon and energy for *M*. *tuberculosis in vivo* is fatty acids. These can be utilized to produce energy through the TCA cycle or, for biosynthesis, by the glyoxylate cycle. It was originally proposed that the importance of isocitrate lyase *in vivo* was due to its role in the glyoxylate cycle [44]. However it has also been recognized that it has additional roles since this enzyme is involved in multiple pathways [45]. It functions as a methylisocitrate lyase making it a key enzyme of the methylcitrate cycle that is involved in the utilization of cholesterol and fatty acids with an odd number of carbons [17]. We suggest an additional role for isocitrate lyase as the first enzyme in a branch point from the glyoxylate and methylcitrate cycles involving alanine dehydrogenase (Fig 1).

In microaerobic NRP-1, isocitrate lyase was induced by *M*. *tuberculosis*, but not malate synthase suggesting that during microaerobic conditions carbon flow increases through alanine dehydrogenase. Activation of the glyoxylate cycle during hypoxia has been detected by isotopic labeling, but while intracellular levels of succinate increased 6.5-fold relative to aerobic conditions, malate levels increased only 1.4-fold [8,46]. Glyoxylate from the glyoxylate cycle and pyruvate, produced from the methylcitrate cycle and glycolysis, would be the substrates for alanine dehydrogenase. Isotope labeling experiments indicated that during hypoxia most of the succinate produced and excreted from the cell was from the reductive branch of the TCA cycle, but a significant amount was produced from the glyoxylate cycle [4].

During active growth of *M*. *tuberculosis*, the key enzymes of the glyoxylate and methylcitrate cycles were optimized for energy and biomass production. Isocitrate lyase, methylisocitrate lyase and malate synthase activities were high while alanine dehydrogenase activities were low. In response to conditions that inhibit aerobic respiration and replication, such as hypoxia or nitric oxide, isocitrate lyase is induced along with alanine dehydrogenase. This has been proposed to play a role in redox balancing during entry into the NRP state [1], and would explain the upregulation of isocitrate lyase without a similar change in malate synthase enzyme levels. As oxygen levels continue to decrease the final shift in metabolism would be to the production of enzymes needed for later reactivation. Isocitrate lyase expression would be downregulated and alanine dehydrogenase further upregulated. This conclusion is in line with our previous identification of three respiratory states of *M*. *tuberculosis* [6] and labeling experiments [4].

The tubercle was long thought to be a static structure preventing the escape of *M*. *tuberculosis*. Now the granuloma is seen as a more dynamic structure with ongoing interactions with the bacilli [47,48]. There may be ongoing cycles of increased oxygen or nitric oxide concentrations that produce nonreplicating states interspersed by growth. The alanine dehydrogenase may play a role during these cycles regulating when it is to the bacteria’s advantage to replicate, and when it is safer to remain in a dormant state. Thus the ability to rapidly initiate growth upon reaeration is essential for the survival of *M*. *tuberculosis*.

## Materials and Methods

### Growth conditions

*M*. *tuberculosis* H37Rv and Erdman strains were from the culture collection of this laboratory. The *M*. *tuberculosis* Δ*ald* mutant RVW7 and its complement were constructed previously [11]. Mycobacterial cultures were grown at 37°C in Dubos Tween-albumin broth (DTA) (Difco, Detroit, Mich). DTA consists of 0.05% (w/v) tryptone, 15 mM asparagine, 153 μM Tween-80, 7 mM KH_2_PO_4_, 18 mM Na_2_HPO_4_, 189 μM ferric ammonium citrate, 83 μM MgSO_4_, 5 μM CaCl_2_, 348 nM ZnSO_4_, 400 nM CuSO_4_, 15 mM NaCl, 0.5% bovine serum albumin fraction V and 42 mM dextrose.

Aerobic cultures were incubated on a model G24 rotary shaker-incubator (New Brunswick Scientific, Edison, N.J.). For hypoxic cultures (Wayne model) slow magnetic stirring in sealed tubes with a headspace ratio of 0.5 was used [18]. After approximately 67 hrs of incubation cultures entered microaerobic NRP-1, and at 200 hrs fully anaerobic NRP-2 was reached. Growth was monitored by following optical density at 580 nm.

DETA/NO was used as the nitric oxide source and conditions were as previously described [19]. All antibiotics and chemicals were from Sigma (St. Louis, MO).

### Construction of *E*. *coli* strain expressing *M*. *tuberculosis ald*

To create the *ald* expressing strain, SZ194, a *ldhA pflB* strain of *E*. *coli*, provided by Lonnie Ingram was used [49]. The alanine dehydrogenase gene from *M*. *tuberculosis* was amplified using primers p204 and p207 (S1 Table). This was cloned into the plasmid pLOI4215 using *Spe*I and *Nsi*I so that *ald* was controlled by the *E*. *coli ldhA* promoter [49]. The resulting plasmid pLOI-AldC was electroporated into SZ194 that also carried the plasmid pKD46. The Red recombinase, expressed from pKD64, resulted in the recombination of *ald* into the *ldhA* Site of SZ194. The presence of the *M*. *tuberculosis ald* in the *ldhA* site was verified by Southern blot. For a control strain a hygromycin marker was also inserted into *ldhA* of SZ194. The insertion of *ald* was verified by PCR. Expression of Ald was verified by Western analysis as previously described [11].

For anaerobic *growth E*. *coli* strains were inoculated into M9 with glucose, and grown in completely filled, sealed tubes. Nitrate when used was at 50 mM.

### Assay of enzyme activities

*M*. *tuberculosis* was grown aerobically to either mid log phase with an approximate OD_580_ of 0.4, to NRP-1 at 115 hrs, or NRP-2 after 250 hrs, in the Wayne model. Cell extracts were prepared using a Mini-Bead Beater (Bio Spec Products, Bartlesville, OK) as previously described [11].

#### Pyruvate and glyoxylate reductive aminase assay

The assay for either pyruvate (PvRA) or glyoxylate (GxRA) reductive aminase activity was based on the oxidation of NADH [11]. Approximately 10 μg of cell extract protein was added to a 1 ml UV transparent cuvette containing 100 mM phosphate buffer. Ammonium sulphate to 400 mM and the substrate (pH 6.4) to 50 mM were added. The reactions were started with the addition of NADH to 80 μM, and measured by the rate of decrease of A_340_ on a Libra S32PC spectrophotometer (Biochrome, Cambridge, UK). A control reaction without ammonium sulphate was included to measure background, and an extinction coefficient of 6220 M^-1^ cm^-1^ was used [50]. Units are μmoles/min/mg protein.

#### Isocitrate lyase

Isocitrate lyase activity was measured as the amount of glyoxylate produced in the presence of phenylhydrazine [51]. The conditions were as previously reported [19]. A control reaction without isocitrate was included to measure background. Units are μmoles/min/mg protein.

#### Malate synthase

The formation of free CoA-SH from acetyl-CoA upon its condensation with glyoxylate was measured [52]. 50 μg of cell extract protein was added to 20 mM Tricine pH 8.0, and 1 mM sodium glyoxylate (pH 6.4). The reaction was started with the addition to 1 mM of acetyl-CoA. At 5 min intervals 50 μl samples were removed and added to 950 μl of 2.5 mM dithiobis nitrobenzoic acid. A control reaction without glyoxylate was included to measure background. The absorbance at 412 nm was measured and an extinction coefficient of 14150 M^-1^ cm^-1^was used [53]. Units are μmoles/min/mg protein.

### Mouse infections

Mouse infections were as reported [19]. 24 C57BL/6 female mice at 8–10 weeks of age (Trudeau Institute) were used for experiments. All procedures involving live animals were performed in the BSL-3 facility in Trudeau Institute in accordance with the Guide for Care and Use of Laboratory Animals of the National Institutes of Health, and individual procedures were approved by the Trudeau Institute Institutional Animal Care and Use Committee. Mice were infected with ~75 CFU of mid-log phase *M*. *tuberculosis* strain H37Rv (Trudeau Mycobacterial Culture Collection no. 102), grown in Proskauer Beck medium containing 0.05% Tween 80, using a Glas-Col airborne infection system, as described [54]. Usually, C57BL/6 mice live between 250 and 300 days post-infection with virulent *M*. *tuberculosis* without apparent signs of disease. Infected animals were carefully monitored during the course of the experiment for evidence of significant pain or extreme distress by the attending veterinarian. Animal technicians monitored feed consumption, fecal output, and behavior of the animal. Three mice were sacrificed by cervical dislocation on day 1 of infection to verify the inoculum in the mouse lungs. Lungs from three mice at days 12, 15, 18, 21, 30, 50 and 100 post-infection were harvested, snap-frozen in liquid nitrogen and kept at −80 °C until use.

### RNA isolation from mouse tissue and quantitative PCR (qRT-PCR)

Mouse infections were as reported [19]. RNA from infected mouse lungs [54] was prepared as described previously. cDNA was produced as reported [11,19]. qRT-PCR on cDNA produced from broth cultures was performed with the Brilliant SYBR Green QPCR Master Mix kit (Stratagene, La Jolla, CA) using an ICycler (Bio-Rad, Hercules, CA) as reported [11]. With cDNA from animal tissue AmpliTaq Gold polymerase (Applied Biosystems) and a Mx400 thermal cycler (Stratagene, Santa Clara, CA) were used [19]. Gene specific *ald* primers and beacons are as described [55]. During mouse lung infection by *M*. *tuberculosis* or in the Wayne model, as levels of 16S rRNA correlated with bacterial CFU numbers [6,54,56].

### Survival and recovery from the Wayne model

For survival during extended hypoxia, wild type and RVW7 were grown in the Wayne model. At intervals tubes were opened for plating on Dubos Oleic Albumin plates and then subsequently discarded.

To analyze recovery from extended hypoxia cultures were grown for 600 hrs (25 days) in the Wayne model. Each tube was then opened and 5 ml of culture was diluted with 5 ml of fresh DTA, followed by aerobic incubation with shaking. Growth was measured by monitoring the OD_580_ or by plating to determine CFUs.

For the competitive assay a spontaneous kanamycin resistant mutant was used as the wild type strain. This wild type at 1.25 x 10^6^ cells/ml was mixed with an equal number of RVW7 cells (hygromycin resistant) in the Wayne model. After 600 hrs the tube was opened and aliquots plated on DOA with either kanamycin (50 μg/ml) or hygromycin (50 μg/ml). The culture was then diluted 1:1 in fresh DTA and grown to mid log phase before being used to set up the next cycle of growth in the Wayne model.

### Metabolic activity

An assay measuring the reducing power of whole cells using menadione as the electron carrier and XTT as the electron acceptor was used [57]. Cultures were grown in the Wayne model for 25 days in DTA. 1 ml of culture was added to a cuvette along with 2,3-bis(2-methoxy-4-nitro-5-sulfenyl)-(2H)-tetrazolium-5-carboxanilide (XTT—to 600 μM) and menadione sodium bisulfate (to 60 μM). The absorbance at 470 nm indicating XTT reduction was determined at 30 min intervals for 3 hrs. The extinction coefficient of XTT is 15600 M^-1^ cm^-1^.

### Determination of the NADH/NAD ratio

Triplicate samples were quickly harvested by centrifuging 2 ml for 2 min. Each pellet was resuspended in 200 μl of either 0.2 M HCl to isolate NAD, or 0.2 M NaOH to isolate NADH. They were then frozen at -80°C until analyzed. Samples were thawed and heated to 80°C for 20 min. They were then cooled and carefully neutralized with Tris buffered 0.2 M HCl or 0.2 M NaOH. It was important to make sure the pH did not go beyond ~7.0 during the neutralization stage since the preparation is based on the selective destruction of either NAD or NADH by pH [58]. Cell debris was pelleted by centrifugation, and the NAD or NADH solutions transferred to new tubes.

To determine NADH or NAD concentrations the nucleotide cycling assay was used [59]. 100 μl aliquots of a reaction buffer containing 167 mM Bicine pH 8.0, 17% ethanol, 7 mM EDTA, 0.7 mM thiazolyl blue tetrazolium bromide and 5.6 mM phenazine ethosulfate were added to a 96 well plate. 90 μl of each NAD or NADH sample was added. The reactions, which were done in triplicate, were started with the addition of 10 μl of alcohol dehydrogenase (5 units). The absorbance at 550 nM was measured at 30 sec intervals for 10 min by a Versa Max Microplate reader (Molecular Devices, Sunnyvale CA). The results were compared to a standard curve. All samples were measured at the same time.

### ATP measurements

ATP was isolated from cultures based on published protocols [18,60]. Briefly 1 ml of washed cells was suspended in HEPES buffer, and an ATP cell extract made by heating at 80°C for 20 min. Triplicate samples consisting of 100 μl of the ATP cell extract was mixed with 50 μl of a solution of luciferin and luciferase, and the light output measured on a Turner model TD-20e Luminometer (Turner Designs, Sunnyvale, CA). Cell densities were determined by measuring CFUs by plating on DOA.

### Methylene blue oxidation

Cultures were grown for 600 hrs in DTA in the Wayne model. Each tube was opened and vortexed to introduce air. Methylene blue (final concentration 31 μM) was added, and 8 ml of culture was transferred to small screw cap tubes which held approximately 8.5 ml. These tubes were sealed and wrapped in parafilm. Cultures were incubated at 37°C with constant mixing and the absorbance at 665 nm measured at intervals.

### Ethics statement

Mouse studies were performed in accordance to the National Institute of Health guidelines using recommendation in the Guide for the Care and Use of laboratory Animals. The protocols used in this study were approved by the Institutional Animal Care and Use Committee of the Trudeau Institute. Animals were sacrificed by cervical dislocation, and all efforts were made to minimize suffering.

## Supporting Information

S1 Fig Hypoxic survival of RVW7 (Δ*ald*). Each strain was grown in the Wayne model and then plated at intervals. WT–circles. RVW7 –squares. The standard deviation is shown.
(TIF)

S2 Fig Anaerobic growth of *E*. *coli* expressing *ald* of *M*. *tuberculosis*. Wild type SZ194 –circles. SZ194 *ldhA*::Hyg with a hygromycin marker in the lactate dehydrogenase gene–squares. SZ194 Mtb *ald*–with the *M*. *tuberculosis ald* expressed from the *E*. *coli ldhA* promoter—triangles.
(TIF)

S1 Table Oligonucleotide primers uses in this study. (PDF)
