# Supplementary material for: Role of Alanine Dehydrogenase of Mycobacterium tuberculosis during Recovery from Hypoxic Nonreplicating Persistence
Source: PLoS One. 2016 May 20;11(5):e0155522. doi: 10.1371/journal.pone.0155522 (PMC4874671; doi:10.1371/journal.pone.0155522)
Supplement: S1 Table — (PDF) [file pone.0155522.s003.pdf]

S1 Table. Oligonucleotide primers uses in this study

Name	Sequence (5'→3')[a]	Purpose in this study[b]
p202	GCGGACGACTGCAAGTTGACCGAGATCATC	qPCR of *ald*
p203	ACCGCAGAATCCTGAGCTTGCTGCATGGCG	qPCR of *ald*
p160	CTGTATCCCGCCAACTCGGTGCCGCAGGTG	qPCR of *icl1*
p161	TCTGCAGCTCGTAGACGTTGAGCGCGCCGC	qPCR of *icl1*
p162	TATCGAGGCGATCTACCTCGGTGGTTGGGC	qPCR of *icl2*
p163	TCGCTCGCTCATCTGCAGGCGTAGATAGTG	qPCR of *icl2*
p164	ATGACGAATGACGCCATCGTCGACACTGAC	qPCR of *glcB*
p165	TAGATGGAGCCGGTGCGGCTGTTGATCAGC	qPCR of *glcB*
p204	TCGAACTTTATGCATACTGAAGCGTACA	Cloning of *ald*
p207	CATCATCGCTTCCACTAGTCCCGACGTGTGC	Cloning of *ald*

[a] Mismatches used to create restriction sites for *ald* cloning are indicated by underlining

[b] qPCR, quantitative reverse transcriptase PCR
